# Perceived Accuracy of Spine-Related Medical Advice From ChatGPT, TikTok, and the North American Spine Society Clinical Practice Guidelines

**DOI:** 10.7759/cureus.88808

**Published:** 2025-07-26

**Authors:** Divya Bhatia, Michael S Kim, Melissa Romoff, Asha Timm, Emily Mills, Hao-Hua Wu, Sohaib Hashmi, Don Park, Yu-Po Lee

**Affiliations:** 1 Department of Research, Palos Verdes High School, Palos Verdes Estates, USA; 2 Department of Orthopedic Surgery, University of California, Irvine, School of Medicine, Orange, USA; 3 Fielding School of Public Health, University of California Los Angeles, Los Angeles, USA

**Keywords:** chatgpt, clinical practice guidelines, patient perception, social media, tiktok

## Abstract

Background: Patients increasingly turn to large language models (LLMs) and social media platforms for medical advice. The accuracy of these sources, particularly compared to peer-reviewed clinical practice guidelines, remains poorly characterized.

Materials and methods: This cross-sectional study evaluated the perceived accuracy of spine-related medical advice generated by ChatGPT (ChatGPT (OpenAI, powered by GPT-4, San Francisco, CA, USA), TikTok (Los Angeles, CA, USA), and the North American Spine Society (NASS) clinical practice guidelines. Medical advice for four spine pathologies was collected from each source. Sixteen orthopedic surgeons rated the accuracy of excerpted recommendations on a 10-point Likert scale. Descriptive statistics summarized mean ratings and standard deviations.

Results: For lumbar stenosis, mean (±SD) accuracy scores were 7.75 ± 2.11 for ChatGPT, 7.00 ± 1.80 for NASS, and 2.50 ± 1.54 for TikTok. For lumbar spondylolisthesis, scores were 7.56 ± 1.50 for ChatGPT, 5.94 ± 2.63 for NASS, and 5.31 ± 2.49 for TikTok. For lumbar disc herniation with radiculopathy, scores were 7.25 ± 2.13 on ChatGPT, 7.06 ± 1.55 on NASS, and 6.44 ± 2.03 on TikTok. For cervical radiculopathy, scores were 7.13 ± 1.38 for ChatGPT, 4.00 ± 2.44 for NASS, and 6.50 ± 2.12 for TikTok.

Conclusions: ChatGPT-generated outputs received the highest ratings for perceived accuracy. NASS guidelines, while evidence-based and peer-reviewed, remain inaccessible to most patients. Professional societies may consider adapting guideline content for dissemination via widely used digital platforms to improve public education and reduce misinformation.

## Introduction

The public increasingly queries large language models (LLMs) and social media platforms for medical advice, yet the accuracy and clinical reliability of these sources remain poorly characterized [1,2]. Although professional society clinical practice guidelines, such as those from the North American Spine Society (NASS), offer comprehensive, evidence-based recommendations, these documents are often inaccessible or difficult to interpret for the general public. As a result, many patients, particularly younger individuals, seek medical information from non-peer-reviewed but easily accessible sources, including social media and LLMs.

TikTok, a video-sharing platform with over one billion active users, has become a significant source of health information, particularly among individuals aged 18 to 30. However, multiple studies have demonstrated that TikTok content frequently contains incomplete or inaccurate medical information, often provided by individuals without appropriate medical expertise [3-5]. Moreover, recent data suggest a direct, positive correlation between video virality and inaccuracy [6].

In parallel, LLMs like ChatGPT (OpenAI, powered by GPT-4, San Francisco, CA, USA) have become widely adopted for their ability to generate detailed and coherent responses to complex queries. Although ChatGPT has demonstrated utility in various domains, including healthcare, concerns remain regarding its potential to produce inaccurate or overly verbose content that may mislead patients without medical training, who may be unaware of and unable to assess the inaccuracy [7-9].

In light of these trends, the accuracy and clinical utility of digital platforms must be systematically assessed. The quality of spine-related medical information generated by ChatGPT and TikTok (Los Angeles, CA, USA) was evaluated and compared to NASS guidelines, which served as the reference standard. Active orthopaedic surgeons were surveyed to assess the perceived accuracy of information generated by these platforms. The study aimed to evaluate the feasibility of integrating these tools into patient education and to identify opportunities for professional societies to improve dissemination of guideline-based content through widely used digital platforms.

## Materials and methods

Study design

This was a cross-sectional study designed to evaluate the perceived accuracy of spine-related medical advice generated by ChatGPT, TikTok, and NASS guidelines.

Data collection

Medical advice on four common spine pathologies, such as cervical radiculopathy, lumbar disc herniation, lumbar spondylolisthesis, and lumbar stenosis, was collected for analysis. Prompts were submitted to ChatGPT using the query format “Give me advice on (condition),” with a new chat opened for each prompt to prevent prior responses from influencing new ones. The responses generated by ChatGPT are included in the appendix. Similarly, TikTok was queried using the phrase “Advice on (condition),” and the first three relevant videos for each condition were reviewed and transcribed. The URLs for each TikTok video are also provided in the appendix. Recommendations for each diagnosis were excerpted from the corresponding NASS guidelines [10-13].

Survey development

Excerpted advice from each source was incorporated into an online survey form. Each participant was blinded to the source of the information. The survey was distributed electronically to 16 orthopedic surgeons. Participants were asked to rate the clinical accuracy of each treatment recommendation using a 10-point Likert scale, where 1 represented entirely inaccurate advice and 10 represented entirely accurate advice.

Statistical analysis

Descriptive statistics were calculated for each condition and information source. An overall comparison between platforms was performed using one-way analysis of variance (ANOVA). Post-hoc pairwise comparisons were conducted using Tukey’s honestly significant difference (HSD) test to evaluate pairwise differences in perceived accuracy between platforms. A p-value < 0.05 was considered statistically significant for all comparisons. All analyses were performed using Python (SciPy and statsmodels packages, Wilmington, DE, USA).

## Results

A total of 16 orthopedic surgeons (aged 26-69 years) responded. Ratings were assigned using a 10-point Likert scale, where higher scores indicated greater perceived accuracy.

For lumbar stenosis, the mean (±SD) accuracy scores were 7.75 ± 2.11 for ChatGPT, 7.00 ± 1.80 for NASS guidelines, and 2.50 ± 1.54 for TikTok. For lumbar spondylolisthesis, mean (±SD) scores were 7.56 ± 1.50 for ChatGPT, 5.94 ± 2.63 for NASS guidelines, and 5.31 ± 2.49 for TikTok. For lumbar disc herniation with radiculopathy, mean (±SD) scores were 7.25 ± 2.13 for ChatGPT, 7.06 ± 1.55 for NASS guidelines, and 6.44 ± 2.03 for TikTok. For cervical radiculopathy, mean (±SD) scores were 7.13 ± 1.38 for ChatGPT, 4.00 ± 2.44 for NASS guidelines, and 6.50 ± 2.12 for TikTok. These scores are presented in Table 1.

**Table 1:** Accuracy ratings by condition and platform SD: standard deviation

Condition	ChatGPT (mean ± SD)	NASS (mean ± SD)	TikTok (mean ± SD)
Lumbar stenosis	7.75 ± 2.11	7.00 ± 1.80	2.50 ± 1.54
Lumbar spondylolisthesis	7.56 ± 1.50	5.94 ± 2.63	5.31 ± 2.49
Lumbar disc herniation	7.25 ± 2.13	7.06 ± 1.55	6.44 ± 2.03
Cervical radiculopathy	7.13 ± 1.38	4.00 ± 2.44	6.50 ± 2.12

An overall comparison across all platforms and conditions was performed using ANOVA. The ANOVA demonstrated a statistically significant difference in accuracy ratings between the three platforms (F = 13.21, p < 0.00001). Post-hoc pairwise analysis using the Tukey HSD test revealed that ChatGPT was rated significantly higher than TikTok (mean difference 2.41, p < 0.001), and NASS guidelines was rated significantly higher than TikTok (mean difference 1.28, p = 0.018). There was no statistically significant difference between ChatGPT and NASS guidelines (mean difference 1.13, p = 0.087). This analysis is presented in Table 2.

**Table 2:** Post-hoc Tukey analysis comparing perceived accuracy between platforms CI: confidence interval, NASS: North American Spine Society

Comparison	Mean difference	95% CI	p-value
ChatGPT vs. NASS	1.125	-0.12 to 2.37	0.087
ChatGPT vs. TikTok	2.406	1.16 to 3.66	<0.001
NASS vs. TikTok	1.281	0.03 to 2.53	0.018

## Discussion

In this study, the perceived accuracy of spine-related medical advice generated by ChatGPT, TikTok, and NASS guidelines was evaluated by orthopedic surgeons. ChatGPT consistently received the highest perceived accuracy ratings, followed by NASS guidelines and TikTok content. Although the NASS guidelines represent evidence-based recommendations developed through peer review and expert consensus, ChatGPT-generated outputs were often rated comparably or more favorably than those from other sources. Ratings for TikTok content demonstrated substantial variability.

Statistical testing confirmed significant differences in perceived accuracy between platforms. ANOVA demonstrated overall differences between sources, and post-hoc testing identified significantly higher accuracy ratings for ChatGPT and NASS guidelines compared to TikTok, with no significant difference between ChatGPT and NASS guidelines. These findings suggest that, for selected clinical topics, ChatGPT may produce advice perceived by physicians as approaching or matching guideline-based accuracy.

The consistency of ratings across conditions also differed between platforms. ChatGPT demonstrated relatively narrow standard deviations, indicating consistent performance across a wide range of queries. In contrast, TikTok exhibited wide variability, likely reflecting its unregulated nature, heterogeneous contributors, and lack of editorial oversight. NASS guidelines demonstrated intermediate variability, potentially reflecting challenges associated with excerpting guideline recommendations for simplified survey evaluation. While NASS guidelines are rigorously developed, they are not optimized for direct patient consumption, which may affect perceived clarity and accuracy when simplified.

These findings are consistent with prior evaluations of medical content quality on digital platforms. Prior studies have demonstrated that popular health information on TikTok often contains substantial inaccuracies, with greater viewership sometimes correlating with misinformation [6,14]. LLMs, such as ChatGPT, have shown potential clinical utility in prior studies; however, their outputs are generated probabilistically and not directly authored or reviewed by clinicians [7,15].

Several limitations warrant consideration. The sample size was limited to 16 orthopedic surgeons, and only four spine conditions were evaluated. TikTok sampling was restricted to the first three videos returned per condition. Ratings reflect physician perceptions of accuracy rather than objective measures of clinical correctness or patient outcomes. Furthermore, public awareness of clinical practice guidelines remains limited, and even when accessible, many patients lack the necessary training to locate and interpret these resources.

These results highlight an opportunity for professional societies to improve the dissemination of high-quality, evidence-based guidance. As patients increasingly seek medical information from LLMs and social media platforms, organizations may improve public education by adapting guideline content into accessible, accurate formats that align with contemporary patterns of digital media consumption.

## Conclusions

This study evaluated the perceived accuracy of spine-related medical advice generated by ChatGPT, TikTok, and NASS guidelines. Orthopaedic surgeons rated ChatGPT outputs most highly, followed by NASS guidelines and TikTok content, though variability existed across conditions. While NASS guidelines are developed through expert consensus and peer-reviewed evidence, they are not widely accessible or easily interpretable by the public. As patients increasingly seek medical information through social media and AI platforms, professional societies may enhance public education by adapting evidence-based guidance into accessible formats for widely used digital channels, thereby mitigating misinformation and improving patient understanding.

## Appendices

ChatGPT-4o response - cervical radiculopathy

Give me advice on cervical radiculopathy.

ChatGPT said:

Of course - let’s go through cervical radiculopathy systematically. Since you didn’t specify whether you want clinical management, surgical indications, or general advice, I’ll structure it broadly but concisely. You can tell me if you want to go deeper into any section:

Cervical radiculopathy: overview and management

Definition

Cervical radiculopathy occurs when a cervical nerve root is compressed or irritated, resulting in neck pain that radiates into the upper extremity, accompanied by sensory, motor, or reflex changes in a dermatomal/myotomal distribution.

Common etiologies

· Cervical disc herniation (soft disc)

· Cervical spondylosis (osteophyte formation, foraminal stenosis)

· Less common: trauma, tumor, infection

Clinical presentation

· Unilateral neck and arm pain

· Sensory paresthesia/numbness

· Motor weakness

· Hyporeflexia (most commonly diminished biceps or triceps reflex)

· Positive Spurling's, Shoulder Abduction Sign, or Lhermitte's sign

Nerve root

Motor

Reflex

Sensory

C5

Deltoid

Biceps

Lateral upper arm

C6

Biceps, wrist extensors

Brachioradialis

Thumb, lateral forearm

C7

Triceps, wrist flexors, finger extensors

Triceps

Middle finger

C8

Finger flexors, intrinsic hand

None

Ring/small finger, medial forearm

Diagnostic workup

· Clinical exam: most important initial tool

· Imaging:

o MRI cervical spine: gold standard

o CT myelogram if MRI contraindicated

· EMG/NCS: if diagnosis is unclear or multiple levels are suspected

Conservative treatment (first-line for most)

· Duration: 6-12 weeks of nonoperative care

· Medications:

o NSAIDs

o Oral corticosteroids (short course)

o Neuropathic agents (gabapentin, pregabalin)

· Physical therapy: cervical stabilization, traction

· Activity modification

· Selective nerve root blocks: diagnostic and therapeutic

Surgical indications

· Progressive neurological deficit

· Persistent disabling pain > 6-12 weeks despite conservative therapy

· Myelopathy (if present, surgical threshold is lower)

· Severe motor weakness

Surgical options

· Anterior cervical discectomy and fusion (ACDF) - most common

· Posterior foraminotomy - motion-preserving, for pure foraminal stenosis

· Cervical disc arthroplasty - selected cases with soft disc herniation and preserved motion

Prognosis

· ~75-90% improve with nonoperative treatment

· Surgical outcomes are generally excellent for well-selected patients

· Long-term recurrence is uncommon but possible

Red flags (urgent eval)

· Signs of myelopathy

· Bilateral symptoms

· Gait instability

· Bowel/bladder dysfunction

· Severe, unrelenting axial neck pain

ChatGPT-4o response - lumbar stenosis

Give me advice on lumbar stenosis.

ChatGPT said:

Here’s a focused overview on lumbar stenosis, framed as practical clinical advice:

Lumbar stenosis: key points

1. Pathophysiology

Central canal narrowing (degenerative disc disease, facet hypertrophy, ligamentum flavum thickening, spondylolisthesis)

Often multilevel, progressive, and associated with degenerative aging

2. Clinical presentation

Neurogenic claudication: classic leg pain, numbness, or weakness that occurs with standing/walking, relieved by sitting or lumbar flexion

Back pain may or may not be prominent

Radiculopathy is possible if foraminal stenosis predominates

3. Differential diagnosis

Peripheral vascular disease

Hip/knee OA

Diabetic neuropathy

Myelopathy (if upper motor neuron signs)

4. Diagnosis

Primarily clinical

MRI: modality of choice - evaluates canal diameter, nerve root compression

CT myelogram: an alternative in MRI-contraindicated patients

EMG is rarely needed except to rule out peripheral neuropathy

5. Nonoperative management (first-line)

Physical therapy: core strengthening, flexion-based exercises

NSAIDs, analgesics: short-term use

Epidural steroid injections: may provide temporary relief, but have limited long-term efficacy

Weight loss, activity modification

6. Indications for surgery

Progressive neurologic deficits

Severe functional limitation despite conservative care

Failure of >3-6 months of nonoperative management

7. Surgical options

Decompression alone (laminectomy): the mainstay for isolated central stenosis

Decompression + fusion: consider if:

Instability (spondylolisthesis, motion on flexion-extension films)

Significant deformity

Severe foraminal stenosis refractory to decompression alone

MIS (minimally invasive surgery) options are increasingly common

8. Prognosis

Generally favorable surgical outcomes

Durable relief of neurogenic claudication

Patients with predominant back pain alone have less predictable results

ChatGPT-4o response - spondylolisthesis

Give me advice on spondylolisthesis.

ChatGPT said:

Of course - here’s a concise, high-yield overview of spondylolisthesis, with practical advice depending on your context (clinical, academic, or surgical):

1. Definition

· Forward slippage of one vertebral body relative to the one below

· Graded by Meyerding classification (Grade I: 0-25% slip, up to Grade V: complete spondyloptosis)

2. Etiology/types

Type

Mechanism

Common location

Isthmic (Lytic)

Pars interarticularis defect (stress fracture)

L5-S1

Degenerative

Facet arthropathy and disc degeneration

L4-L5

Dysplastic

Congenital dysplasia of posterior elements

L5-S1

Traumatic

Acute fracture other than pars

Variable

Pathologic

Tumor, infection, bone disease

Variable

Postsurgical

Iatrogenic instability

Variable

3. Symptoms

· Axial low back pain

· Radiculopathy (nerve root compression)

· Neurogenic claudication (if associated with stenosis)

· Rarely, cauda equina syndrome

4. Workup

· Imaging:

o Plain radiographs (standing lateral flexion/extension views)

o MRI (neural elements, stenosis, disc)

o CT (pars defects)

· Grading: Meyerding scale

· Stability assessment: flexion-extension films for surgical planning

5. Nonoperative management (first-line in most cases)

· NSAIDs, physical therapy, activity modification

· Core strengthening/McKenzie exercises

· Epidural steroid injections for radicular pain

· Brace (rarely indicated long-term)

6. Operative management

Indications:

· Failed conservative management

· Progressive slip or deformity

· Neurologic deficits

· Severe stenosis

Surgical options:

Procedure

Indication

Posterior decompression alone

Low-grade stable slips, isolated stenosis

Posterolateral fusion (PLF)

Low to moderate grade slips

TLIF/PLIF

More common for stability, indirect decompression

ALIF

Especially at L5-S1 (good access to disc, lordosis restoration)

Instrumented fusion

For instability, high-grade slips

High-grade spondylolisthesis (>50% slip):

· Consider reduction vs in situ fusion carefully

· Watch for L5 nerve root stretch injury during reduction

7. Prognosis

· Many patients with low-grade slips can be managed nonoperatively

· Surgical outcomes are generally excellent for appropriately selected patients

· High-grade slips carry a higher surgical risk but can still have good long-term outcomes with appropriate technique

8. Key pearls

· Always correlate radiographic findings with symptoms

· High-grade slips require careful preoperative planning to avoid nerve root injury

· Consider spinopelvic parameters (pelvic incidence, sacral slope) for surgical planning

· In degenerative spondylolisthesis, indirect decompression with interbody fusion may preserve segmental alignment and improve outcomes

ChatGPT-4o response - lumbar disc herniation

Give me advice on lumbar disc herniation.

ChatGPT said:

Here’s a concise, structured summary of lumbar disc herniation (LDH) and its management, as would be appropriate for an orthopaedic spine surgeon or academic audience:

Lumbar disc herniation: overview and management

Pathophysiology

· Displacement of nucleus pulposus through an annulus fibrosus defect

· Most commonly at L4-L5 and L5-S1 levels

· Mechanical compression + chemical irritation of adjacent nerve root → radiculopathy

Clinical presentation

· Unilateral radicular leg pain (sciatica) may have back pain

· Neurological findings depending on level:

o L4: Anterior thigh pain, weak quadriceps, diminished patellar reflex

o L5: Dorsiflexion weakness, sensory changes over the dorsum of the foot

o S1: Plantarflexion weakness, diminished Achilles reflex, lateral foot numbness

· Red flags: Cauda equina syndrome (saddle anesthesia, bowel/bladder dysfunction, severe bilateral symptoms)

Diagnosis

· History and physical exam are primary

· MRI of the lumbar spine is the gold standard imaging

· Electrodiagnostic studies (EMG/NCS) may assist in equivocal cases

Natural history

· The majority improve with conservative management

· Spontaneous resorption of herniated disc material is well-documented (immunologically mediated macrophage activity)

Nonoperative management

· First-line for most:

o Activity modification

o NSAIDs/analgesics

o Short course of oral steroids (controversial)

o Physical therapy (flexion exercises, core strengthening)

o Epidural steroid injections (selective nerve root block may aid both diagnosis and symptom control)

· Typically trialed for 6-12 weeks unless severe/progressive deficits

Surgical indications

· Progressive neurological deficit

· Severe intractable radicular pain refractory to conservative care

· Cauda equina syndrome (urgent decompression)

· Failure of nonoperative treatment after ~6-12 weeks

Surgical options

· Microdiscectomy (standard of care):

o Minimally invasive; high success rates (>90% relief of radiculopathy)

o Complications: Dural tear, infection, recurrent herniation (~5-10%)

· Endoscopic discectomy: increasingly used at select centers

· Fusion is rarely indicated for isolated LDH unless instability or recurrent herniation with segmental pathology

Prognosis

· Excellent for isolated radiculopathy

· Predictors of favorable surgical outcomes: shorter symptom duration, younger age, and predominant leg pain over back pain

Key literature

· SPORT trial (Weinstein et al., NEJM, 2006): surgery provides faster pain relief, but long-term outcomes are similar to nonoperative care in many cases

· Recent meta-analyses continue to support early surgery for faster recovery in appropriately selected patients

TikTok videos

Cervical Radiculopathy

1. https://www.tiktok.com/@dr.manbun_pt/video/7219305755101515050?is_from_webapp=1&sender_device=pc&web_id=7515976653224117806

2. https://www.tiktok.com/@panagiotispissalidis/video/6965863722795486470?is_from_webapp=1&sender_device=pc&web_id=7515976653224117806

3. https://www.tiktok.com/@isabelle.spt/video/7384860782577634603?is_from_webapp=1&sender_device=pc&web_id=7515976653224117806

Lumbar Stenosis

1. https://www.tiktok.com/@pittsburgh_chiropractor/video/6907397261395807494?is_from_webapp=1&sender_device=pc&web_id=7515976653224117806

2. https://www.tiktok.com/@irvinespine/video/7186692983822224686?is_from_webapp=1&sender_device=pc&web_id=7515976653224117806

3. https://www.tiktok.com/@doctorhoeflinger/video/7515156414105586990?is_from_webapp=1&sender_device=pc&web_id=7515976653224117806

Spondylolisthesis

1. https://www.tiktok.com/@donnallyspineconsult/video/7268466428473265450?is_from_webapp=1&sender_device=pc&web_id=7515976653224117806

2. https://www.tiktok.com/@backinshapeprogram/video/7198542211124874501?is_from_webapp=1&sender_device=pc&web_id=7515976653224117806

3. https://www.tiktok.com/@drchollkim/video/7351917272165911850?is_from_webapp=1&sender_device=pc&web_id=7515976653224117806

Lumbar Disc Herniation

1. https://www.tiktok.com/@squatuniversity/video/7150323999854087467?is_from_webapp=1&sender_device=pc&web_id=7515976653224117806

2. https://www.tiktok.com/@corycalendinemd/video/7385174127960739118?is_from_webapp=1&sender_device=pc&web_id=7515976653224117806

3. https://www.tiktok.com/@getadjustednow/video/7451640121171954986?is_from_webapp=1&sender_device=pc&web_id=7515976653224117806

## References

[1] Dagher T, Dwyer EP, Baker HP, Kalidoss S, Strelzow JA (2024). “Dr. AI will see you now”: how do ChatGPT-4 treatment recommendations align with orthopaedic clinical practice guidelines?. Clin Orthop Relat Res.

[2] Kirkpatrick CE, Lawrie LL (2024). TikTok as a source of health information and misinformation for young women in the United States: survey study. JMIR Infodemiology.

[3] Tolson H, Wenger D, McCune M, Alzahrani F, Dupuy E (2023). TikTok as a source of medical information: port wine stain birthmarks and treatment using vascular lasers. Cureus.

[4] Birkun AA (2024). Misinformation on first aid for seizures communicated through the fastest growing social media platform: a cross-sectional study of TikTok content. Epilepsy Behav.

[5] Kolade O, Martinez R, Awe A, Dubin JM, Mehran N, Mulcahey MK, Tabaie S (2023). Misinformation about orthopaedic conditions on social media: analysis of TikTok and Instagram. Cureus.

[6] Subramanian T, Araghi K, Akosman I (2023). Quality of spine surgery information on social media: a discern analysis of TikTok videos. Neurospine.

[7] Romoff M, Brunette M, Peterson MK, Hashmi SZ, Kim MS (2025). The role of large language models in improving the readability of orthopaedic spine patient educational material. J Orthop Surg Res.

[8] Walker HL, Ghani S, Kuemmerli C, Nebiker CA, Müller BP, Raptis DA, Staubli SM (2023). Reliability of medical information provided by ChatGPT: assessment against clinical guidelines and patient information quality instrument. J Med Internet Res.

[9] Nastasi AJ, Courtright KR, Halpern SD, Weissman GE (2023). A vignette-based evaluation of ChatGPT's ability to provide appropriate and equitable medical advice across care contexts. Sci Rep.

[10] Hoang T, Liou L, Rosenberg AM (2024). An analysis of ChatGPT recommendations for the diagnosis and treatment of cervical radiculopathy. J Neurosurg Spine.

[11] North American Spine Society (2010). North American Spine Society. Diagnosis and Treatment of Cervical Radiculopathy from Degenerative Disorders. Clinical practice guideline. North American Spine Society. Diagnosis and Treatment of Cervical Radiculopathy From Degenerative Disorders. Clinical Practice Guideline.

[12] North American Spine Society (2011). North American Spine Society. Diagnosis and Treatment of Degenerative Lumbar Spinal Stenosis. Clinical practice guideline. North American Spine Society. Diagnosis and Treatment of Degenerative Lumbar Spinal Stenosis. Clinical Practice Guideline.

[13] North American Spine Society (2014). North American Spine Society. Diagnosis and Treatment of Degenerative Lumbar Spondylolisthesis. Clinical practice guideline. North American Spine Society. Diagnosis and Treatment of Degenerative Lumbar Spondylolisthesis. Clinical Practice Guideline.

[14] North American Spine Society (2012). North American Spine Society. Diagnosis and Treatment of Lumbar Disc Herniation with Radiculopathy. Clinical practice guideline. North American Spine Society. Diagnosis and Treatment of Lumbar Disc Herniation With Radiculopathy. Clinical Practice Guideline.

[15] Rao SJ, Isath A, Krishnan P (2024). ChatGPT: a conceptual review of applications and utility in the field of medicine. J Med Syst.

